# Excessive Erythrocytosis and Chronic Mountain Sickness in Dwellers of the Highest City in the World

**DOI:** 10.3389/fphys.2020.00773

**Published:** 2020-07-15

**Authors:** Ivan Hancco, Sébastien Bailly, Sébastien Baillieul, Stéphane Doutreleau, Michèle Germain, Jean-Louis Pépin, Samuel Verges

**Affiliations:** ^1^HP2 Laboratory, Univ. Grenoble Alpes, INSERM, Grenoble Alpes University Hospital, Grenoble, France; ^2^Laboratoire Interuniversitaire de Biologie de la Motricité (LIBM) EA7424, Team “Vascular Biology and Red Blood Cell”, Université Claude Bernard Lyon 1, University of Lyon, Lyon, France

**Keywords:** hypoxia, altitude, red blood cell, hematocrit, chronic mountain sickness

## Abstract

**Background:**

While millions of people are living permanently at high altitude (>2,500 m) worldwide, the mechanisms underlying their tolerance to chronic hypoxia and those responsible for the occurrence of chronic mountain sickness (CMS) remain to be elucidated. Excessive erythrocytosis (EE) is thought to be the main mechanism responsible for CMS symptoms and is included in the definition of CMS, but the precise interplay between EE and symptoms of CMS requires further investigations.

**Methods:**

The present study benefits from an exceptional dataset coming from 1,594 dwellers of La Rinconada, the highest city in the world (5,100–5,300 m). Based on individual clinical characteristics, subjects were categorized according to the presence of EE and CMS diagnosis, based on current guidelines.

**Results:**

In this population of relatively young [32 (23; 39) years] highlanders residing in La Rinconada for only a few years [3 (2; 5) years], the internal prevalence of EE (44%) was high, whereas the internal prevalence of CMS (14%) was similar compared to previous reports in highlander populations living at lower altitude (∼4,000 m) in the Andes. Individuals with EE reported less symptoms compared to individuals with lower hematocrit values. Multivariable analysis revealed that age and sex are the main factors associated with EE, whereas age, hematocrit and number of years living at La Rinconada are factors associated with CMS symptoms.

**Conclusion:**

In this specific population of La Rinconada, high hematocrit values were observed but were associated with limited symptoms. These results raise important questions regarding the definition of EE and CMS and their underlying mechanisms in high-altitude populations.

## Introduction

Approximately 140 million individuals reside at high altitude (>2,500 m) worldwide, the largest populations of highlanders being found in South America (Andean), central Asia (Tibetan and Sherpa), and East Africa (Ethiopian) (West, 2017). Chronic mountain sickness (CMS) is a clinical syndrome observed in 5 to 33% of individuals residing permanently at high altitude (Leon-Velarde et al., 2005). It has long been recognized especially in Andean populations (Monge, 1928, 1943) and frequently terminates in cardiorespiratory diseases including pulmonary hypertension and right- or left-sided heart failure. The current international consensus statement suggests that the diagnosis of CMS should be based on an elevated hemoglobin concentration [Hb] (≥21 g/dL for males or ≥19 g/dL for females) and a minimum score based on the following symptoms: breathlessness and/or palpitations, sleep disturbances, cyanosis, dilatation of veins, paresthesia, headaches, and tinnitus (Leon-Velarde et al., 2005). Clinical observations indicate, however, that many highlanders show high [Hb] but no symptoms, whereas others report symptoms without [Hb] reaching the above thresholds (Gonzales et al., 2013).

These observations have led to consider excessive erythrocytosis (EE) as a specific pathophysiological entity or a preclinical form of CMS in individuals residing permanently at high altitude (Vargas and Spielvogel, 2006). The optimal [Hb] at a given altitude and the definition of EE currently based on [Hb] thresholds provided by the recommendations for CMS diagnosis (Leon-Velarde et al., 2005) are still a matter of debate (Villafuerte et al., 2004; Vargas and Spielvogel, 2006). First, polycythemia is essentially a required adaptive response to long-term hypoxic exposure in order to preserve tissue oxygen delivery and is not a hallmark of high-altitude maladaptation. Second, the main stimulus for erythrocytosis is hypoxemia, which depends on the altitude level, and therefore threshold values to determine EE may depend on the altitude of residence. Current recommendations for EE in highlanders use threshold [Hb] values corresponding to 2 SDs above the mean [Hb] observed in healthy young highlanders living close to 4,000 m [(Vasquez and Villena, 2001), 4,000 m; (Monge et al., 1989), 4,300 m]. Highlanders with [Hb] above these thresholds are considered at risk of developing maladaptations to chronic hypoxic exposure and CMS symptoms. However, whether these thresholds for EE and their expected consequences on health status also apply to highlanders at different altitudes remains an important issue to clarify.

Chronic hypoxic exposure at high altitude can induce respiratory, cardiovascular, and neurological dysfunctions even in the absence of EE (Naeije and Vanderpool, 2013; Yan, 2014; Latshang et al., 2017), owing to oxidative stress and sympathetic overactivation for instance (Zhuang et al., 1993; Bailey et al., 2013). Therefore, in individuals living permanently at high altitude, symptoms such as breathlessness, sleep disturbances, palpitations, or headaches may occur, indicating some degree of maladaptation to chronic hypoxic exposure despite the absence of EE.

Hence, there is a need for large-scale observational studies of populations permanently residing at high altitude to better characterize their health status and especially the relationship between hypoxemia, [Hb], and symptoms considered to be associated with CMS. La Rinconada in Peru is the highest city in the world [5,100–5,300 m (West, 2017)] with approximately 70,000 inhabitants (according to the 2016 census, INEI-Peru) mostly involved in gold mining activities. The present study investigates for the first time a large sample of people living in La Rinconada in order to disentangle the relationship between hypoxemia, erythrocytosis, and symptoms in highlanders. We hypothesized that EE and symptoms of CMS represent two entities at least partly independent.

## Materials and Methods

### Study Population

**FIGURE 1:**
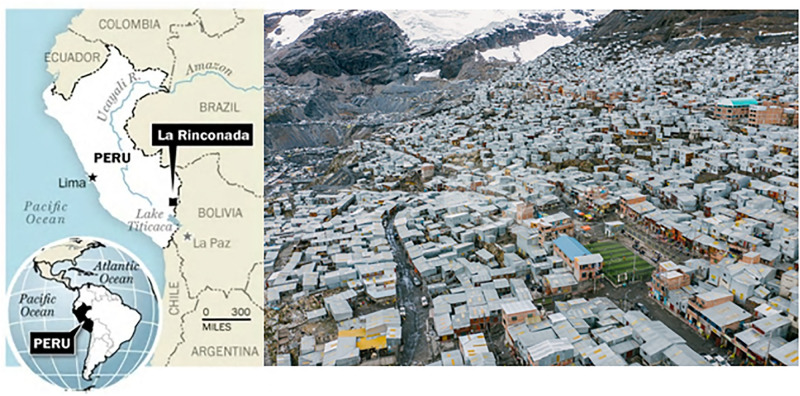
Localization of the highest city in the World, La Rinconada, Peru (5,100–5,300 m). Adapted from Enserink (2019).

We evaluated 1,594 adults who volunteered to be part of the present study between January 2016 and July 2017. Subjects were between 18 and 57 years old and permanent residents of La Rinconada, located between 5,100 and 5,300 m of altitude in the San Antonio de Putina province, south of Peru (Figure 1). Subjects were recruited during a medical consultation destined to families of miners working in a gold mine facility in La Rinconada, and all data were anonymized prior analysis. All subjects had at least 1 year of residency in La Rinconada. The study was approved by the Ethics Committees of Inter-région Rhône-Alpes-Auvergne (IRB-5891) and Universidad Nacional Mayor de San Marcos (CIEI-2019-002).

### Data Collection

Information about age, sex, ethnic group, duration of residence at La Rinconada, and socioeconomic status were obtained during a medical consultation conducted by a native Spanish speaker medical doctor (I. H.) for routine medical follow-up of miners and their family in La Rinconada. Chronic mountain sickness was diagnosed through the current CMS scoring system including seven symptoms of CMS according to the Qinghai questionnaire and the evaluation of EE (Leon-Velarde et al., 2005). After at least 5 min of rest in a sitting position under temperate room conditions (16–18°C), pulse oxygen saturation (SpO_2_) and heart rate were recorded from the finger (NELLCOR OxiMaxN-65; Tyco Healthcare, Pleasanton, CA, United States). The hematocrit was measured manually (microhematocrit) from a blood sample obtained at the fingertip. The systolic and diastolic blood pressures were determined using manual sphygmomanometer.

### EE and CMS Classifications

Based on current recommendations to characterize CMS (Leon-Velarde et al., 2005) and previous studies having investigated EE and CMS symptoms in highlanders, subjects were classified according to three distinct classifications:

1. Classification 1 [EE based on the current international consensus statement (Leon-Velarde et al., 2005)]: subjects with or without EE, that is, with a hematocrit level ≥63 or <63% for males and ≥57 or <57% for females, respectively.
2. Classification 2 (EE calculated based on the present population): because the threshold for EE based on the current consensus has been defined from a young healthy group of Andeans living in Cerro de Pasco [4,300 m, (Monge et al., 1989)], that is, approximately 1,000 m below La Rinconada, we intended to calculate a new threshold for EE based on the present population from La Rinconada according to the method used in Cerro de Pasco by Monge et al. (1989). We have calculated this threshold as the mean + 2 SD hematocrit value of young healthy subjects (i.e., 18–25 years, ≤2 symptoms in the Qinghai questionnaire); thereafter, subjects were classified as with or without EE.
3. Classification 3 [current recommendations for CMS diagnosis (Leon-Velarde et al., 2005)]: subjects with no, mild, moderate, or severe total CMS score (based on the scores of the seven symptoms described above summed with the hematocrit score, defined based on EE according to Classification 1), that is, with a total CMS score ≤5 or no EE, between 6 and 10 with EE, between 11 and 14 with EE, or ≥15 with EE, respectively.

### Statistical Analysis

Qualitative data were expressed as frequency and percentages, and quantitative variables as median and interquartile range. Comparisons between groups within each classification were performed using χ^2^ test for qualitative variables and Kruskal–Wallis test for quantitative ones. Groups or item categories with low sample size were gathered together for analysis. A two-tailed α level of 0.05 was used as the cutoff for significance. Correlations between quantitative variables were assessed using Spearman coefficient. To identify the factors associated with EE [according to the current consensus (Leon-Velarde et al., 2005)] and CMS symptoms (according to the seven symptoms from the Qinghai questionnaire), a univariable analysis using a logistic regression model was performed. Variables with a *p* value threshold of 0.20 in the univariable analysis were selected and were introduced in a multivariable logistic regression model. A stepwise selection was performed to identify the final model. All statistical procedures were performed on SASv9.4 (SAS Inc., Cary, NC, United States) by S. Bailly and S. Verges.

### Role of the Funding Source

The funding sources had no role regarding study design; the collection, analysis, and interpretation of data; writing of the manuscript; and decision to submit it for publication.

## Results

### Whole Population

Characteristics of the whole population are provided in Table 1. Symptoms of CMS, hematocrit score, and total CMS score for the whole population are provided in Table 2. Table 1 shows the distribution of subjects according to the three different classifications.

**TABLE 1:** Description of subject characteristics for the whole population.

	Median [IQR] or n (%)
Age (years)	32 [23; 39]
*Sex*	
Female	235 (14.7)
Male	1,359 (85.3)
*Ethnic group*	
Aymara	75 (4.7)
Quechua	1,519 (95.3)
Residency in La Rinconada (years)	3 [2; 5]
Hematocrit (%)	60 [54; 66]
Heart rate (bpm)	87 [75; 94]
SpO_2_ (%)	82 [78; 85]
Diastolic blood pressure (mm Hg)	70 [70; 80]
Systolic blood pressure (mm Hg)	100 [100; 110]
**Classification 1 (excessive erythrocytosis, international consensus)**	
No excessive erythrocytosis	891 (55.9)
Excessive erythrocytosis	703 (44.1)
**Classification 2 (excessive erythrocytosis, calculated threshold)**	
No excessive erythrocytosis	1,381 (86.6)
Excessive erythrocytosis	213 (13.4)
**Classification 3 (total CMS score, international consensus)**	
≤5 (no CMS)	1,373 (86.1)
6–10 (mild total CMS score)	156 (9.8)
11–14 (moderate total CMS score)	52 (3.3)
>14 (severe total CMS score)	13 (0.8)

*CMS, chronic mountain sickness; IQR, interquartile range; SpO_2_, pulse oxygen saturation. Classifications 1 and 3 were performed according to the current international consensus on CMS (Leon-Velarde et al., 2005), while Classification 2 was performed based on a calculated threshold for excessive erythrocytosis in the population from La Rinconada (see section “Materials and Methods” for further details).*

**TABLE 2:** Symptoms of CMS, hematocrit score, and total CMS score in the whole population.

	Median [IQR] or n (%)
**Breathlessness/palpitations**	
0	1,230 (77.2)
1	211 (13.2)
2	145 (9.1)
3	8 (0.5)
**Sleep disturbance**	
0	1,290 (80.9)
1	284 (17.8)
2	20 (1.3)
3	0 (0)
**Cyanosis**	
0	1,178 (73.9)
1	280 (17.6)
2	136 (8.5)
3	0 (0)
**Dilatation of veins**	
0	1,328 (83.3)
1	181 (11.4)
2	64 (4)
3	21 (1.3)
**Paresthesia**	
0	1,222 (76.7)
1	293 (18.4)
2	72 (4.5)
3	7 (0.4)
**Headache**	
0	15 (0.9)
1	1,203 (75.5)
2	321 (20.1)
3	55 (3.5)
**Tinnitus**	
0	183 (11.5)
1	1,161 (72.8)
2	198 (12.4)
3	52 (3.3)
**Hematocrit**	
0	891 (55.9)
3	703 (44.1)
**Total CMS score**	5 [3; 6]

*IQR, interquartile range; CMS, chronic mountain sickness.*

**FIGURE 2:**
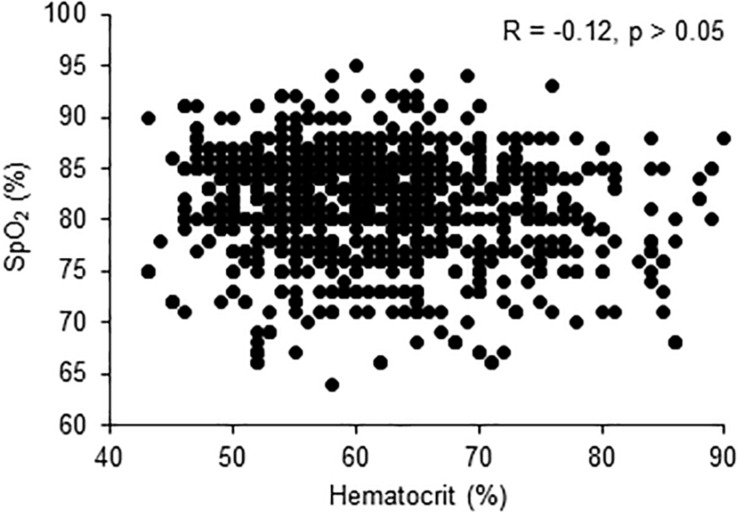
Correlation between pulse oxygen saturation (SpO_2_) and hematocrit levels.

There were no significant correlations between hematocrit and SpO_2_ (Figure 2) and between hematocrit or SpO_2_ and any other quantitative variable (all Spearman correlation coefficient <0.50).

#### Factors Associated With EE [According to the Current International Consensus (Leon-Velarde et al., 2005)]

**FIGURE 3:**
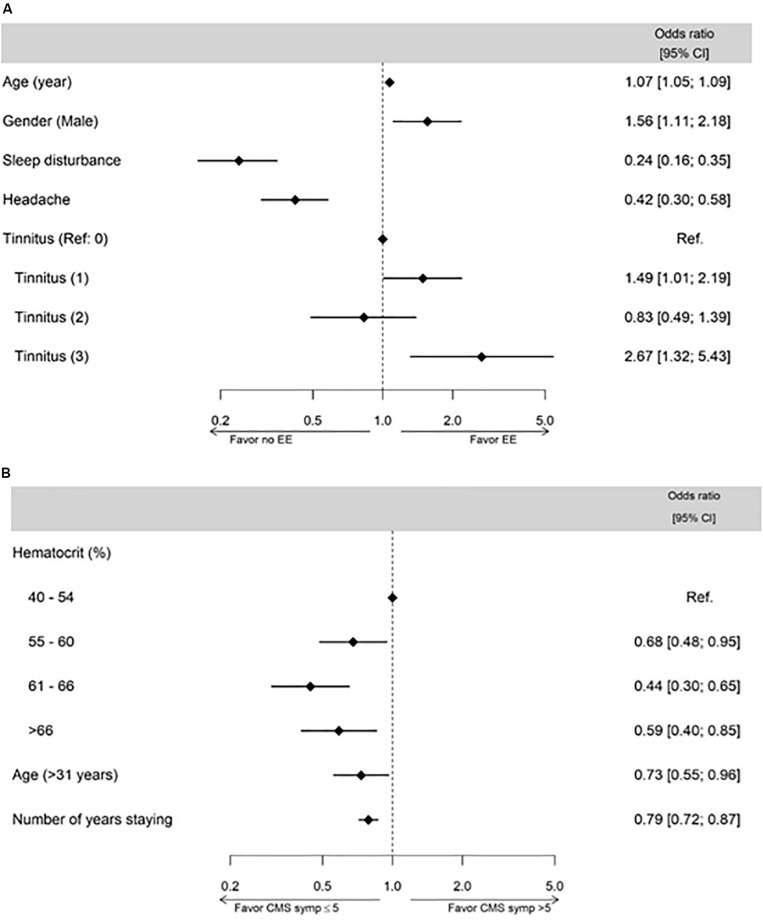
Subject physiological characteristics and symptoms associated with excessive erythrocytosis (EE **(A)** and CMS symptoms **(B)** assessed by multivariable linear regression model analysis.

Multivariable logistic regression revealed that the factors associated with EE were age, sex, symptoms of sleep disturbances, headaches, and tinnitus (Figure 3A). Factors associated with an increased probability of having EE were older age, being male, and tinnitus scores 1 and 3. Symptoms of sleep disturbances and headaches were associated with a decreased probability of having EE.

#### Factors Associated With CMS Symptoms (According to the 7 Symptoms From the Qinghai Questionnaire)

Multivariable logistic regression revealed that the factors associated with CMS symptoms score were age, number of years residing in La Rinconada, and hematocrit (Figure 3B). Older age (>31 years), a higher number of years staying in La Rinconada, and a higher hematocrit were associated with a decreased probability of having CMS symptoms score >5.

### Description of the CMS Classifications

#### Classification 1 (Tables 3, 4)

In males, all variables except ethnic group, heart rate, blood pressure, and paresthesia were significantly different between both groups. Subjects with EE were older, stayed in La Rinconada for a longer duration, and had a lower SpO_2_ and a higher hematocrit (by definition) compared to subjects without EE. Males with EE reported less severe CMS symptoms than males without EE.

**TABLE 3:** Comparison of male characteristics according to erythrocytosis profiles [Classification 1, current international consensus (Leon-Velarde et al., 2005)].

	Without EE n (%) or median [IQR]	With EE n (%) or median [IQR]
n (%)	720 (53.0)	639 (47.0)
Age (years)	27 [23; 36]	36 [27; 42]*
*Ethnic group*		
Aymara	33 (4.6)	29 (4.5)
Quechua	687 (95.4)	610 (95.5)
Residency in La Rinconada (years) (years)	3 [2; 5]	3 [3; 5]*
Hematocrit (%)	56 [52; 60]	68 [65; 74]*
SpO_2_ (%)	82 [80; 85]	81 [78; 85]*
Heart rate (bpm)	85 [75; 93]	87 [74; 94]
Diastolic blood pressure (mm Hg)	70 [70; 80]	70 [70; 80]
Systolic blood pressure (mm Hg)	100 [100; 110]	100 [100; 110]
**Breathlessness/palpitations**		
0	519 (72.1)	527 (82.5)*
1	113 (15.7)	67 (10.5)*
2–3	88 (12.2)	45 (7)*
**Sleep disturbance**		
0	535 (74.3)	592 (92.6)*
1–3	185 (25.7)	47 (7.4)*
**Cyanosis**		
0	499 (69.3)	515 (80.6)*
1	149 (20.7)	84 (13.1)*
2–3	72 (10)	40 (6.3)*
**Dilatation of veins**		
0	572 (79.4)	553 (86.5)*
1	104 (14.4)	55 (8.6)*
2	36 (5)	21 (3.3)*
3	8 (1.1)	10 (1.6)*
**Paresthesia**		
0	538 (74.7)	497 (77.8)
1	136 (18.9)	118 (18.5)
2–3	46 (6.4)	24 (3.8)
**Headache**		
0	502 (69.7)	551 (86.2)*
1–3	218 (30.3)	88 (13.8)*
**Tinnitus**		
0	96 (13.3)	47 (7.4)*
1	488 (67.8)	526 (82.3)*
2	115 (16)	42 (6.6)*
3	21 (2.9)	24 (3.8)*

EE, excessive erythrocytosis; IQR, interquartile range; SpO_2_, pulse oxygen saturation. Scores of symptom severity were grouped when the number of subjects per score was too small to allow statistical analysis. * Significantly different from subjects without EE (*p* < 0.05).

**TABLE 4:** Comparison of female characteristics according to erythrocytosis profiles [Classification 1, current international consensus (Leon-Velarde et al., 2005)].

	Without EE n (%) or median [IQR]	With EE n (%) or median [IQR]
n (%)	171 (72.8)	64 (27.2)
Age (years)	25 [21; 35]	24.5 [22; 32]
*Ethnic group*		
Aymara	10 (5.8)	3 (4.7)
Quechua	161 (94.2)	61 (95.3)
Residency in La Rinconada (years) (years)	3 [2; 5]	4 [3; 5]
Hematocrit (%)	50 [49; 54]	60 [58; 63]*
SpO_2_ (%)	83 [80; 85]	82 [80; 85]
Heart rate (bpm)	90 [79; 97]	86.5 [71.5; 93]*
Diastolic blood pressure (mm Hg)	70 [70; 80]	70 [70; 80]
Systolic blood pressure (mm Hg)	100 [100; 110]	100 [100; 105]
**Breathlessness/palpitations**		
0	124 (72.5)	60 (93.8)*
1	29 (17)	2 (3.1)*
2–3	18 (10.5)	2 (3.1)*
**Sleep disturbance**		
0	99 (57.9)	64 (100)*
1–3	72 (42.1)	0 (0)*
**Cyanosis**		
0	107 (62.6)	57 (89.1)*
1	44 (25.7)	3 (4.7)*
2–3	20 (11.7)	4 (6.3)*
**Dilatation of veins**		
0	141 (82.5)	62 (96.9)*
1	20 (11.7)	2 (3.1)*
2	7 (4.1)	0 (0)*
3	3 (1.8)	0 (0)*
**Paresthesia**		
0	128 (74.9)	59 (92.2)*
1	34 (19.9)	5 (7.8)*
2–3	9 (5.3)	0 (0)*
**Headache**		
0	103 (60.2)	62 (96.9)*
1–3	68 (39.8)	2 (3.1)*
**Tinnitus**		
0	36 (21.1)	4 (6.3)*
1	87 (50.9)	60 (93.8)*
2	41 (24)	0 (0)*
3	7 (4.1)	0 (0)*

EE, excessive erythrocytosis; IQR, interquartile range; SpO_2_, pulse oxygen saturation. Scores of symptom severity were grouped when the number of subjects per score was too small to allow statistical analysis. * Significantly different from subjects without EE (*p* < 0.05).

In females, only hematocrit, heart rate, and symptoms were significantly different between both groups. Females with EE reported less severe CMS symptoms than females without EE.

#### Classification 2 (Tables 5, 6)

The mean + 2 SD hematocrit values in young healthy subjects (males, *n* = 220; females, *n* = 72) from the present population were 72% for males and 68% for females. These values were used to define EE within Classification 2, that is, ≥72% for males and ≥68% for females.

**TABLE 5:** Comparison of male characteristics according to erythrocytosis profiles (Classification 2, calculated threshold).

Variable	Without EE n (%) or median [IQR]	With EEn (%) or median [IQR]
n (%)	1,150 (84.6)	209 (15.4)
Age (years)	31.5 [23; 38]	38 [34; 45]*
*Ethnic group*		
Aymara	49 (4.3)	13 (6.2)
Quechua	1,101 (95.7)	196 (93.8)
Residency in La Rinconada (years)	3 [2; 5]	3 [3; 5]
Hematocrit (%)	60 [55; 65]	75 [74; 77]*
Spo_2_ (%)	82 [78; 85]	81 [77; 84]*
Heart rate (bpm)	87 [75; 94]	82 [73; 90]*
Diastolic blood pressure (mm Hg)	70 [70; 80]	70 [70; 80]
Systolic blood pressure (mm Hg)	100 [100; 110]	100 [100; 120]
**Breathlessness/palpitations**		
0	873 (75.9)	173 (82.8)
1	161 (14.0)	19 (9.1)
2–3	116 (10.1)	17 (8.1)
Sleep disturbance		
0	931 (81.0)	196 (93.8)*
1–3	219 (19.0)	13 (6.2)*
**Cyanosis**		
0	853 (74.2)	161 (77.0)
1	198 (17.2)	35 (16.7)
2–3	99 (8.6)	13 (6.2)
**Dilatation of veins**		
0	949 (82.5)	176 (84.2)*
1	134 (11.7)	25 (12.0)*
2	55 (4.8)	2 (1.0)*
3	12 (1.0)	6 (2.9)*
**Paresthesia**		
0	871 (75.7)	164 (78.5)
1	222 (19.3)	32 (15.3)
2–3	57 (5.0)	13 (6.2)
**Headache**		
0	870 (75.7)	183 (87.6)*
1–3	280 (24.3)	26 (12.4)*
**Tinnitus**		
0	127 (11.0)	16 (7.7)*
1	841 (73.1)	173 (82.8)*
2	146 (12.7)	11 (5.3)*
3	36 (3.1)	9 (4.3)*

EE, excessive erythrocytosis; IQR, interquartile range; SpO_2_, pulse oxygen saturation. Scores of symptom severity were grouped when the number of subjects per score was too small to allow statistical analysis. * Significantly different from subjects without EE (*p* < 0.05).

**TABLE 6:** Comparison of female characteristics according to erythrocytosis profiles (Classification 2, calculated threshold).

Variable	Without EE n (%) or median [IQR]	With EE n (%) or median [IQR]
n (%)	231 (98.3)	4 (1.7)
Age (years)	25 [22; 33]	22.5 [22; 23]
*Ethnic group*		
Aymara	49 (4.3)	13 (6.2)
Quechua	1,101 (95.7)	196 (93.8)
Residency in La Rinconada (years)	3 [2; 5]	3.5 [2; 4.5]
Hematocrit (%)	53 [49; 57]	70 [69; 73]
Spo_2_ (%)	83 [80; 85]	75.5 [72.5; 78]
Heart rate (bpm)	90 [77; 96]	78 [66; 93.5]
Diastolic blood pressure (mm Hg)	70 [70; 80]	75 [70; 80]
Systolic blood pressure (mm Hg)	100 [100; 110]	100 [100; 100]
**Breathlessness/palpitations**		
0	180 (77.9)	4 (100)
1	31 (13.4)	0 (0)
2–3	20 (8.7)	0 (0)
**Sleep disturbance**		
0	159 (68.8)	4 (100)
1–3	72 (31.2)	0 (0)
Cyanosis		
0	161 (69.7)	3 (75)
1	46 (19.9)	1 (25)
2–3	24 (10.4)	0 (0)
**Dilatation of veins**		
0	199 (86.1)	4 (100)
1	22 (9.5)	0 (0)
2	7 (3)	0 (0)
3	3 (1.3)	0 (0)
**Paresthesia**		
0	185 (80.1)	2 (50)
1	37 (16)	2 (50)
2–3	9 (3.9)	0 (0)
**Headache**		
0	161 (69.7)	4 (100)
1–3	70 (30.3)	0 (0)
**Tinnitus**		
0	38 (16.5)	2 (50)
1	145 (62.8)	2 (50)
2	41 (17.7)	0 (0)
3	7 (3)	0 (0)

*EE, excessive erythrocytosis; IQR, interquartile range; SpO_2_, pulse oxygen saturation. Scores of symptom severity were gathered together when the number of subjects per score was too small to allow statistical analysis. No statistical comparison has been done because of the small number of subjects with EE.*

In males, all variables except ethnic group, duration of residency in La Rinconada, blood pressure, symptoms of breathlessness/palpitations, cyanosis, and paresthesia were significantly different between both groups. Subjects with EE were older, had a lower SpO_2_ and heart rate, and a higher hematocrit (by definition) compared to subjects without EE. Males with EE reported less severe sleep disturbance, dilatation of veins, headache, and tinnitus than males without EE.

In females, only 1.7% of the subjects presented EE according to the calculated threshold, making statistical comparison between groups with and without EE inappropriate.

#### Classification 3 (Tables 7, 8)

In males, all variables except ethnic group and blood pressures were significantly different between groups. Males with no CMS spent a larger number of years in La Rinconada that males with mild total CMS score. The hematocrit was, by definition, significantly higher in males with CMS and even higher in males with moderate total CMS score compared to males with mild total CMS score, respectively. SpO_2_ was significantly lower in males with mild total CMS score compared to males with no CMS and moderate and severe total CMS score. Heart rate was significantly higher in males with mild total CMS score compared to males with no CMS. The severity of breathlessness and palpitations, cyanosis, paresthesia, and headache was higher in males with mild, moderate, and severe total CMS score compared to males with no CMS. The severity of dilatations of veins and tinnitus was significantly increased only in males with moderate and severe total CMS score compared to males with no CMS and mild total CMS score. The severity of sleep disturbance was significantly increased only in males with severe total CMS score compared to the other groups.

**TABLE 7:** Comparison of male characteristics according to total CMS score (Leon-Velarde et al., 2005).

	No CMS (≤5) n (%) or median [IQR]	Mild total CMS score (6–10) n (%) or median [IQR]	Moderate total CMS score (11–14) n (%) or median [IQR]	Severe total CMS score (>14) n (%) or median [IQR]
n (%)	1,146 (84)	149 (11.0)	55 (4.0)	9 (1.0)
Age (years)	32 [24; 39]	34 [26; 45]*	33 [23; 43]	40 [33; 40]
*Ethnic group*				
Aymara	53 (4.6)	7 (4.7)	1 (1.8)	1 (11.1)
Quechua	1,093 (95.4)	142 (95.3)	54 (98.2)	8 (88.9)
Residency in La Rinconada (years)	3 [2; 5]	3 [2; 4]*	3 [2; 4]	2 [2; 3]
Hematocrit (%)	60 [55; 65]	67 [65; 73]*	70 [68; 75]* ^,+^	63 [63; 75]* ^,+^
Arterial oxygen saturation (%)	82 [79; 85]	80 [78; 82]*	83 [78; 85]^+^	83 [83; 83]^+^
Heart rate (bpm)	85 [74; 94]	88 [80; 96]*	88 [72; 92]	92 [78; 92]
Diastolic blood pressure (mm Hg)	70 [70; 80]	70 [70; 80]	70 [70; 80]	70 [70; 80]
Systolic blood pressure (mm Hg)	100 [100; 110]	100 [100; 110]	100 [90; 110]	100 [100; 110]
**Breathlessness/palpitations**				
0	943 (82.3)	103 (69.1)*	0 (0)*	0 (0)*
1	115 (10)	32 (21.5)*	24 (43.6)*	9 (100)*
2–3	88 (7.7)	14 (9.4)*	31 (56.4)*	0 (0)*
**Sleep disturbance**				
0	961 (83.9)	118 (79.2)	48 (87.3)	0 (0)* ^,+,^^[*dollar*]^
1–3	185 (16.1)	31 (20.8)	7 (12.7)	9 (100)* ^,+,^^[*dollar*]^
**Cyanosis**				
0	1,074 (93.7)	123 (82.6)*	41 (74.5)* ^,+^	9 (100)* ^,+^
1–3	72 (6.3)	26 (17.4)*	14 (25.5)* ^,+^	0 (0)* ^,+^
**Dilatation of veins**				
0	1,102 (96.2)	143 (96)	30 (54.5)* ^,+^	9 (100)* ^,+^
1–3	44 (3.8)	6 (4)	25 (45.5)* ^,+^	0 (0)* ^,+^
**Paresthesia**				
0	941 (82.1)	85 (57)*	9 (16.4)* ^,+^	0 (0)* ^,+,^^[*dollar*]^
1	159 (13.9)	55 (36.9)*	40 (72.7)* ^,+^	0 (0)* ^,+,^^[*dollar*]^
2–3	46 (4)	9 (6)*	6 (10.9)* ^,+^	9 (100)* ^,+,^^[*dollar*]^
**Headache**				
0	928 (81)	102 (68.5)*	23 (41.8)* ^,+^	0 (0)* ^,+^
1–3	218 (19)	47 (31.5)*	32 (58.2)* ^,+^	9 (100)* ^,+^
**Tinnitus**				
0	1,010 (88.1)	128 (85.9)	19 (34.5)* ^,+^	0 (0)* ^,+,^^[*dollar*]^
1–3	136 (11.9)	21 (14.1)	36 (65.5)* ^,+^	9 (100)* ^,+,^^[*dollar*]^
**Hematocrit**				
0	720 (62.8)	0 (0)*	0 (0)*	0 (0)*
3	426 (37.2)	149 (100)*	55 (100)*	9 (100)*

IQR, interquartile range; CMS, chronic mountain sickness. Scores of symptom severity were grouped when the number of subjects per score was too small to allow statistical analysis. * Significantly different from subjects with no CMS. ^+^Significantly different from subjects with mild total CMS score. ^$^Significantly different from subjects with moderate total CMS score (*p* < 0.05).

**TABLE 8:** Comparison of female characteristics according to total CMS score (Leon-Velarde et al., 2005).

	No CMS (≤5) n (%) or median [IQR]	Mild total CMS score (6–10) n (%) or median [IQR]	Moderate total CMS score (>10) n (%) or median [IQR]
n (%)	227 (96.6)	7 (3.0)	1 (0.4)
Age (years)	25 [22; 33]	23 [22; 33]	26 [26; 26]
*Ethnic group*			
Aymara	12 (5.3)	1 (14.3)	0 (0)
Quechua	215 (94.7)	6 (85.7)	1 (100)
Residency in La Rinconada (years)	3 [2; 5]	3 [2; 4]	6 [6; 6]
Hematocrit (%)	53 [49; 56]	61 [57; 67]*	60 [60; 60]
SpO_2_ (%)	83 [80; 85]	80 [76; 83]	83 [83; 83]
Heart rate (bpm)	90 [76; 97]	90 [88; 94]	93 [93; 93]
Diastolic blood pressure (mm Hg)	70 [70; 80]	70 [70; 80]	60 [60; 60]
Systolic blood pressure (mm Hg)	100 [100; 110]	100 [100; 110]	90 [90; 90]
**Breathlessness/palpitations**			
0	180 (79.3)	4 (57.1)	0 (0)
1	29 (12.8)	1 (14.3)	1 (100)
2–3	18 (7.9)	2 (28.6)	0 (0)
**Sleep disturbance**			
0	155 (68.3)	7 (100)	1 (100)
1–3	72 (31.7)	0 (0)	0 (0)
**Cyanosis**			
0	207 (91.2)	4 (57.1)*	0 (0)
1–3	20 (8.8)	3 (42.9)*	1 (100)
**Dilatation of veins**			
0	217 (95.6)	7 (100)	1 (100)
1–3	10 (4.4)	0 (0)	0 (0)
**Paresthesia**			
0	181 (79.7)	6 (85.7)	0 (0)
1	37 (16.3)	1 (14.3)	1 (100)
2–3	9 (4)	0 (0)	0 (0)
**Headache**			
0	159 (70)	6 (85.7)	0 (0)
1–3	68 (30)	1 (14.3)	1 (100)
**Tinnitus**			
0	179 (78.9)	7 (100)	1 (100)
1–3	48 (21.1)	0 (0)	0 (0)
**Hematocrit**			
0	171 (75.3)	0 (0)*	0 (0)
3	56 (24.7)	7 (100)*	1 (100)

IQR, interquartile range; CMS, chronic mountain sickness; SpO_2_, pulse oxygen saturation. Scores of symptom severity were gathered together when the number of subjects per score was too small to allow statistical analysis. * Significantly different from subjects with no CMS (*p* < 0.05). No statistical comparison was performed with subject with moderate-severe total CMS score because of small sample size; no subject presented severe total CMS score.

In females, only eight subjects (3.4%) had CMS. Only hematocrit and symptoms of cyanosis differed between groups. Females with no CMS had a lower hematocrit and less severe cyanosis than female with mild total CMS score.

## Discussion

This is the first study to provide physiological and clinical characteristics of a large sample of highlanders living in the highest city in the world, La Rinconada, at 5,100 to 5,300 m of altitude. The internal prevalence of EE (44%) according to the current international consensus is high in this population compared to previous observations in highlanders living at lower altitude. Conversely, the internal prevalence of CMS (14%) is relatively similar compared to previous observations in populations living at lower altitude (∼4,000 m). Interestingly, the results from this specific population of relatively young highlanders suggest a dissociation between the presence of EE and the presence of CMS symptoms. Individuals with EE had less severe CMS symptoms than individuals without EE, and conversely, individuals reporting CMS symptoms had a lower hematocrit than individuals without CMS symptoms. This study provides unique insights into the physiological and clinical characteristics of highlanders living in the highest city in the world and raises important issues regarding the definition of EE and CMS in highlanders.

### Whole Population Characteristics

The population investigated in the present study comprises relatively young individuals, with a large majority of males. This can be explained by the fact that people in La Rinconada mostly stay in this city for professional reasons (to work in the gold mine), and the individuals evaluated in the present study are employees or family members of gold mine employees. The majority of the subjects resided in La Rinconada for a few years only, but all originate from villages and cities >3,800 m in the Puno region. Therefore, they represent a specific population of native highlanders exposed at some point during their lifetime to a greater hypoxic stress (i.e., altitude >5,000 m) for several years. This is not a population-based study, and as a consequence, it should be emphasized that the present results cannot apply to the overall population of La Rinconada.

Mean SpO_2_ [mean (SD): 81% (5%)] is lower than values previously reported in relatively large group of Peruvian highlanders living in Cerro de Pasco at 4,340 m [87% in 103 males (Gonzales et al., 2011)], which most probably reflects the lower inspiratory oxygen partial pressure at higher altitude. Only one previous study reported hematological parameters in Andean highlanders (*n* = 273) living above 5,000 m in La Rinconada (Leon-Velarde et al., 2000). Mean hematocrit levels were similar both in adult males (60 vs. 62%) and females (55 vs. 54%) compared to the present study. The normal systemic blood pressure measured in the overall population is in accordance with previous observations in healthy highlanders at lower altitude (Richalet et al., 2005; Corante et al., 2018). The main CMS symptoms reported in the population of La Rinconada are headache and tinnitus, suggesting that chronic hypoxic exposure may be primarily associated with neurological disorders in highlanders living at this extreme altitude (Thomas et al., 2000; Bao et al., 2017).

In the highest Andean population studied previously in Cerro de Pasco (4,340 m), the prevalence of EE defined as an [Hb] above the mean + 2 SD value measured in young healthy subjects was 15.4% in males between 30 and 39 years and reached 33% by the sixth decade of age (Monge et al., 1989), which is well below the 44% (47% in males) prevalence observed overall in the present population. Because the threshold to define EE according to the current international consensus is based on a population of highlanders living at 4,340 m, that is, at a substantially lower altitude than La Rinconada, we intended to calculate a threshold to define EE in the present population of La Rinconada as previously done in Cerro de Pasco. This calculation led to high thresholds for hematocrit values defining EE (i.e., ≥72% for males and ≥68% for females) and consequently to low prevalence of EE according to these new thresholds (i.e., 15.4% for males and 1.7% for females, Tables 5, 6). Although it may seem reasonable to use different thresholds to define EE in populations of highlanders living at different altitudes (from 2,500 to above 5,000 m), the current international consensus on chronic high-altitude diseases provides a single threshold for defining EE in populations living permanently above 2,500 m (Leon-Velarde et al., 2005). In addition, one could consider that if [Hb] ≥ 21 g/dL for males and [Hb] ≥ 19 g/dL for females lead to increased risks of cardiovascular dysfunctions in highlanders living close to 4,000 m (e.g., in Cerro de Pasco), similar risks should be expected in highlanders living at higher altitudes. Therefore, we believe that the relatively low internal prevalence of EE based on the calculated threshold from the healthy young subjects in the population of La Rinconada does not reflect the actual burden of EE and its clinical consequences in this population.

In regions of the central Andes of Peru (>4,000 m), the prevalence of CMS diagnosis has been reported to be 15 to 20% in the adult male population (Villafuerte, 2015). Despite the large prevalence of EE, CMS diagnosis was confirmed in only 14% (15.7% in males) of the population. This relatively low prevalence of CMS could be due to the young age of the present population that may exhibit EE but not yet its pathophysiological consequences (e.g., cardiovascular dysfunctions). Prevalence of CMS has been shown indeed to increase in older highlanders [e.g., >40 years, (Monge et al., 1989; Leon-Velarde et al., 1993)]. This low internal prevalence may also result from the fact that subjects with EE reported few CMS symptoms according to the Qinghai questionnaire and therefore did not reach a Qinghai score > 5 confirming CMS diagnosis.

### Excessive Erythrocytosis and CMS Symptoms

The lower severity of CMS symptoms in subjects with EE compared to subjects without EE is an interesting result from the present study. This result is obtained both when using the definition of EE based on the current international consensus [Classification 1 (Leon-Velarde et al., 2005)] and based on the calculated thresholds from young healthy subjects in La Rinconada (Classification 2). The multivariate logistic regressions determining factors associated with EE and CMS symptoms also confirm the inverse relationship between the presence of EE and the severity of CMS symptoms in the present population (Figure 3). This is in contrast to the traditional conception of CMS as being the consequence of an excessive erythropoiesis, which increases blood viscosity and ultimately leads to symptoms characterizing CMS (Leon-Velarde et al., 2005). This conception is supported, for instance, by the effects of hemodilution in highlanders alleviating CMS symptoms (Cruz et al., 1979; Winslow et al., 1985). The apparent distinction between EE and the occurrence of CMS symptoms in the present population may be the consequence of several factors: (i) the relatively young age and short time of residency in La Rinconada of the subjects who may exhibit EE but not yet CMS symptoms; (ii) subjects with EE resided for a longer duration in La Rinconada and may represent the subjects who were able to tolerate living above 5,000 m without excessive symptoms, whereas the others may have left the city prematurely; (iii) high hematocrit may be an inevitable response to permanent residency above 5,000 m, whereas other mechanisms (e.g., pulmonary arterial hypertension) may underlie CMS symptoms even in subjects without very high hematocrit values.

Systemic blood pressure was similar in subjects with or without EE, as well as in subjects with and without CMS diagnosis, which does not support an increased cardiovascular risk (at least of systemic hypertension) in highlanders with EE or CMS symptoms. Although CMS is generally thought to potentially induce cardiovascular dysfunctions (Leon-Velarde et al., 2005), large sets of data regarding cardiovascular comorbidities in highlanders with and without CMS are still missing (Corante et al., 2018). High hematocrit and no CMS symptoms were associated with older age, which may suggest either that these individuals were more likely to have stayed for a longer period in La Rinconada and therefore have developed appropriate mechanisms to tolerate chronic hypoxic exposure (including tolerance to high hematocrit), or that with aging highlanders may be more prone to develop EE as previously suggested (Monge et al., 1989; Leon-Velarde et al., 1993). The lower prevalence of EE and CMS in females than in males is consistent with previous results showing that female highlanders are less likely to develop high-altitude diseases (Leon-Velarde et al., 2005), possibly due to hormonal differences (Ou et al., 1994). Because postmenopausal females are more likely to present EE and CMS than their premenopausal counterparts (Leon-Velarde et al., 1997), the young age of the present female population also probably led to a reduced prevalence of EE and CMS. Although hypoxemia was slightly but significantly more severe in subjects with EE compared to subjects without EE as previously reported (De Ferrari et al., 2017; Corante et al., 2018), there was no significant correlation between hematocrit and hypoxemia (Figure 2), suggesting that other factors than reduced blood oxygenation *per se* may promote hematological changes (Bermudez et al., 2020). The lower number of years spent in La Rinconada in subjects with mild CMS symptoms and no EE may suggest either that tolerance to chronic hypoxia may require a prolonged period of exposure or that subjects with CMS symptoms may have left La Rinconada prematurely. The cross-sectional design of the study does not allow, however, to clarify this issue, and longitudinal investigations are required to better characterize the adaptation and health issues associated with permanent hypoxic exposure above 5,000 m.

### Limitations

It should be first emphasized that this is not a population-based sample randomly selected from the population of La Rinconada but instead a population of relatively young miners and their family residing in La Rinconada for a few years only and consulting for a medical check-up. Therefore, the present results cannot be generalized to the overall population of La Rinconada but provide useful insight into the physiological characteristics and symptoms of highlanders exposed at some point during their lifetime to very high altitude. It should also be acknowledged that most of the individuals in the present population worked in the gold mines, which could have potential pathophysiological consequences (Vearrier and Greenberg, 2011; Gibb and O’Leary, 2014), in addition to chronic hypoxia *per se*. Males and females have distinct professional activities in La Rinconada (i.e., only males work within the mine, while females work outside) which could be a factor explaining at least in part the difference in prevalence of EE and CMS between males and females. Body mass index has been shown to be a significant risk factor for CMS [e.g., (Julian et al., 2013; De Ferrari et al., 2014)], but this measurement was unfortunately unavailable in the present dataset. While the present study mostly focused on criteria defining CMS (hematocrit and symptoms), further physiological investigations (e.g., pulmonary arterial pressure, ventilatory response, vascular function, sleep recordings, etc.) should be performed in future studies to better understand the mechanisms underlying EE and CMS in the unique population of La Rinconada.

## Conclusion

This study reports, for the first time, the physiological characteristics and symptoms of a large population of highlanders living in the highest city in the world. At >5,000 m of altitude in La Rinconada, the internal prevalence of EE (44%) was substantially greater than previously reported in similar Andean populations living permanently at altitudes ∼1,000 m lower. A striking observation was the dissociation between the presence of EE and the presence of CMS symptoms; that is, individuals with EE reported few symptoms, whereas symptomatic individuals had lower hematocrit compared to asymptomatic counterparts. This may suggest that threshold Hb values to define excessive levels at this altitude should be reconsidered. Furthermore, it suggests that high hematocrit values might be required to reside in La Rinconada with limited symptoms. While the present results apply to a population of relatively young subjects living for some years only in La Rinconada, population-based epidemiological studies, longitudinal studies, and additional physiological investigations including ventilatory responses, cardiovascular function, sleep studies, and exercise testing are required to better clarify the mechanisms underlying EE and CMS symptoms.

## Data Availability Statement

All datasets generated for this study are included in the article/supplementary material.

## Ethics Statement

The studies involving human participants were reviewed and approved by the Ethics Committees of Inter-région Rhône-Alpes-Auvergne. Committee waived the requirement for written informed consent for participants in this study due to the retrospective analysis of anonymized data collected during clinical practice, in accordance with the national legislation and the institutional requirements.

## Author Contributions

All authors contributed to the conception and design of the work, to acquisition, analysis or interpretation of the data, drafted the manuscript, and approved the final version of the manuscript.

## Conflict of Interest

The authors declare that the research was conducted in the absence of any commercial or financial relationships that could be construed as a potential conflict of interest.
